# Is There a Novel Biosynthetic Pathway in Mice That Converts Alcohol to Dopamine, Norepinephrine and Epinephrine?

**DOI:** 10.3390/molecules27092726

**Published:** 2022-04-23

**Authors:** Paul J. Fitzgerald

**Affiliations:** Department of Psychiatry, University of Michigan, Ann Arbor, MI 48109, USA; pfitz1940@gmail.com

**Keywords:** ethanol, catecholamines, substance abuse, dependence, addiction, reward

## Abstract

Previous studies in animals and humans have shown multiple types of interaction between alcohol (ethanol) intake and the catecholamine signaling molecules: dopamine, norepinephrine and epinephrine. This literature suggests that the administration of alcohol to rodents affects the central and peripheral (blood plasma) levels of these catecholamines. Two prior publications (Fitzgerald 2012, 2020) put forth the hypothesis that there may be a currently unidentified biosynthetic pathway, in a range of organisms, that actually converts alcohol to dopamine, norepinephrine and epinephrine. This publication describes the details for how to test this hypothesis in mice. Mice can be systemically injected with an intoxicating dose of commercially available stable isotope-labeled ethanol (ethanol-1-^13^C), and blood plasma samples and brains can be collected approximately two to 24 h post-injection. Liquid chromatography-mass spectrometry analysis can then be used to test whether some of the labeled ethanol molecules have been incorporated into new dopamine, norepinephrine, and epinephrine molecules, in plasma and brain samples. If confirmed, this hypothesis may have broadly reaching implications both for basic neuroscience and our understanding of alcohol abuse and alcoholism.

Alcohol (ethanol) remains a widely used drug throughout much of the world, and is associated with a number of social norms and customs, some of which date back centuries [1,2,3]. Alcohol abuse and dependence also remain widespread public health problems, and are a major source of morbidity and mortality worldwide [4,5]. The pathophysiological processes that underlie the problematic use of this drug are beginning to be elucidated, and growing evidence suggests that, among various factors, alcohol intake interacts with the catecholaminergic neurotransmitters dopamine (DA) and norepinephrine (NE) [6,7]. DA in particular has been widely implicated in the rewarding effects of various drugs, including alcohol [8], and a smaller literature in both animals and humans also implicates NE in the rewarding properties of drugs such as alcohol [9,10]. NE, along with the related molecule epinephrine (EPI; also known as adrenaline), has already been strongly implicated in the dysregulation of stress-related brain circuits that underlies alcohol abuse and alcoholism [11]. Thus, these three catecholaminergic signaling molecules may play a prominent role in the physiological consequences of the long-term use of alcohol.

While moderate to heavy use of alcohol is associated with a number of serious health conditions, such as major depressive disorder and cardiovascular disease, the light consumption of alcohol has been controversially suggested by some researchers to actually have health benefits, including sustained antidepressant properties or improved cognition [12,13,14]. If light use of alcohol really does have some beneficial effects on long-term health, one possibility is that these effects are mediated by alterations in neurotransmitter systems such as NE and DA. Studies of how alcohol modulates noradrenergic signaling in particular, carried out in rodents, suggest a number of important effects. For example, ethanol intake or administration to rats has been shown to increase plasma or brain NE [15,16] including in the medial prefrontal cortex [17], although other studies suggest decreases in NE [18,19]. The voluntary consumption of ethanol by Sardinian alcohol-preferring rats can produce antidepressant-like effects in the forced swim test [20]. Another study of (ethanol naïve) alcohol-preferring rats showed that these animals have elevated cortical NE [21]. Finally, the noradrenergic transmission reducing drugs propranolol (non-selective beta blocker) and prazosin (alpha1 antagonist), reduce operant alcohol-reinforced responding during acute withdrawal in rodents [22,23], and the alpha1 antagonist doxazosin can attenuate drinking in alcohol-dependent humans [24].

Two theoretical publications have suggested an even more direct relationship between alcohol intake and the catecholamines: there may be a novel biosynthetic pathway, present in a wide range of organisms including rodents and humans, that *converts* alcohol to DA, NE, and EPI, possibly by reacting with an unidentified phenylic molecule [25,26]. (This novel pathway is proposed to exist in addition to the canonical pathway for catecholaminergic biosynthesis described over 70 years ago (Figure 1A), discussed below.) These two papers suggest that the existence of such a pathway or pathways could have wide ranging implications for our understanding of alcohol abuse and alcoholism, as well as the basic biochemistry underlying the endogenous synthesis of these important neurotransmitters and signaling molecules. Thus, this theoretical framework provides a rationale for experimentally investigating whether alcohol is converted to these catecholamines in vivo.

The current publication describes an approach for experimentally investigating this overarching hypothesis by combining stable isotope biochemistry with liquid chromatography-mass spectrometry. (In vivo microdialysis could also be combined with this approach to study brain physiology after systemic administration of labeled ethanol to mice.) It is suggested here that this biotransformation to catecholamines takes place rapidly (i.e., possibly within minutes and at least within an hour or two), where such transformation could play a role in the acute psychotropic and intoxicating properties of ethanol. Male (and female) CD-1 mice, an outbred strain, can be studied for blood plasma and homogenized brain tissue including the brainstem, or also in a more regionally specific manner. The mice can be fed normal laboratory rodent chow *ad libitum*. In particular, it is suggested that at least 10 mice be injected with carbon 13 (C13) labeled ethanol, while an additional group of 10 or more control mice are injected with regular carbon 12 (C12) unlabeled ethanol (see below for more details). A time delay of two hours between injection and euthanasia can be used for study, while other animals can be tested with longer time delays, say up to 24 h or longer. (It is also possible that shorter delays, such as 30 min or one hour, may yield greater measured conversion of C13 labeled ethanol into catecholamines). It is suggested here that using unlabeled C12 ethanol mice as the control group, where all other variables are the same (i.e., injection stress, acute intoxication) as in the C13 labeled group, is critical for having a sensitive way to examine the C12/C13 ratio for DA, NE, and EPI in blood plasma or brain samples. In this scenario, an unpaired two-tailed *t*-test (although the argument can be made for an unpaired one-tailed *t*-test) would be a straightforward way to compare the 10 data points (i.e., C12/C13 ratios) from the C12 unlabeled ethanol mice with the 10 points from the C13 labeled ethanol animals, where a significantly lower C12/13 ratio in the C13 mice would be consistent with the existence of a novel biosynthetic pathway leading from ethanol to that neurotransmitter. Also, a mass spectrometer operated in targeted mode could be used to create graphs in order to illustrate if there is a significant peak for C13 labeled dopamine, norepinephrine or epinephrine, in animals that received C13 labeled ethanol. 

Here are some more details about the labeled and unlabeled ethanol. Stable isotopically-labeled C13 ethanol (ethanol-1-^13^C, catalog # 324523) and unlabeled C12 ethanol (catalog # 459844) (MilliporeSigma, Burlington, MA, USA) can each be diluted in water to prepare separate labeled and unlabeled 20% ethanol solutions (*v*/*v*). This stable isotope ethanol is not radioactive and has the same toxicity and other properties as regular (unlabeled) ethanol. All mice can be injected intraperitoneally (i.p.) at a volume of 10 mL/kg, with either ethanol solution at an intoxicating dose of 1.5 g/kg ethanol (i.e., a 30 g mouse would get a 0.3 mL injection volume of 20% ethanol solution). Both types of ethanol can be stored at room temperature, and can be diluted immediately prior to injection. See Figure 1B for the molecular structure of the C13 labeled and C12 unlabeled ethanol proposed in this study.

One criticism of the overall experimental approach proposed here is that perhaps ethanol, like many organic molecules consumed in the diet, could simply be metabolized into its component parts and end up being converted to many molecules, including catecholamines. As diagrammed in my previous publication [26], it is proposed that both carbons in the ethanol molecule are together incorporated into dopamine, where the hydroxyl group of ethanol is converted to an amine. This means that ethanol is not simply broken down into simpler parts that could eventually be converted to catecholamines. This hypothesis could be tested through various stable isotope experiments: (1) Label both hydrocarbons in the same molecule of ethanol (either using C13 or deuterium) to test whether this whole portion of the ethanol molecule is retained in labeled catecholamines. (2) Investigate whether other biological molecules, such as various amino acids, are NOT labeled after presentation of stable isotope ethanol to mice. This experiment would test the specificity of the conversion of labeled ethanol to catecholamines. (3) Inject mice with a stable isotope small molecule or molecules other than ethanol (such as the amino acids glycine or proline) and then verify that these molecules are NOT converted to labeled catecholamines, further testing specificity.

If ethanol can be acutely converted to catecholamines in vivo, it would be highly informative to also measure the quantities of these neurotransmitters that are synthesized and released following alcohol exposure. This would provide information as to whether the potentially direct conversion is of physiological relevance. If such conversion takes place to a significant degree, it may suggest that these enzymatic processes are druggable targets for alcohol abuse and alcoholism, as stated below. While measuring the potential biosynthesis of catecholamines in whole brain homogenate may yield coarse information on this process, a preferable approach would be to dissect different brain regions, such as the locus coeruleus, to better localize regional specificity. Perhaps this could also be done in mice pretreated with the drug reserpine that then have minimal amounts of monoaminergic neurotransmitters stored in vesicles. As noted above, these approaches could be combined with microdialysis.

The only significant known endogenous pathway for the biosynthesis of the three catecholamines comprises the following pathway: tyrosine (a non-essential amino acid) -> L-DOPA -> DA -> NE -> EPI (Figure 1A), which was identified over 70 years ago [27,28,29,30]. The hypothesis described here suggests that ethanol reacts with an unidentified (possibly phenylic) molecule or molecules (to form DA, possibly independently of tyrosine and L-DOPA) [26]. As described in that previous publication, some candidate phenylic molecules, present in various plants and dietary sources, are shown here in Figure 1C. As mentioned above, in this scenario ethanol would attach to the benzene ring structure of these molecules and then the hydroxyl group of ethanol would be converted to an amine, yielding dopamine. As also noted in Figure 1C, quercetin and catechin already contain an ethanol-like moiety (shown in yellow) which, even in the absence of ethanol, may help convert these two flavonoids to dopamine through endogenous, currently unidentified enzymatic processes. In these scenarios, DA could then be converted to NE and then EPI through the aforementioned canonical pathway described over 70 years ago [26]. Another possibility is that there are unidentified, *independent* pathways that convert ethanol to DA, NE, or EPI, separately. In either case, if such a pathway or pathways exist, the enzyme or enzymes that catalyze these novel reactions remain to be identified, along with the anatomical locations (such as the locus coeruleus in the brainstem or its axonal terminal projections in the cortex, and perhaps the adrenal gland) and cell types in the body where these reactions occur.

If there is a biosynthetic pathway (or pathways) that converts alcohol to DA, NE, and EPI in vivo, in mammals and other organisms, then this could have broadly reaching implications for the prevention and treatment of alcohol abuse and alcoholism. For example, genetic, epigenetic, or environmentally-induced variability in these catecholaminergic systems may predispose some individuals to alcoholism to maintain a relatively high “set point” or “tone” for these systems, for example [25,26]. Similarly, since NE, EPI, and also DA can be released centrally or peripherally in response to psychological stress [31,32,33], the craving or relapse of problematic drinking in response to stress may be partly based in replenishing depleted cellular stores of these three molecules [25,26]. In this scenario, pharmacological treatments targeting these putative pathways (including their potentially novel enzymes, when identified) may have therapeutic properties with regard to preventing or treating alcohol abuse and alcoholism.

## Figures and Tables

**Figure 1:**
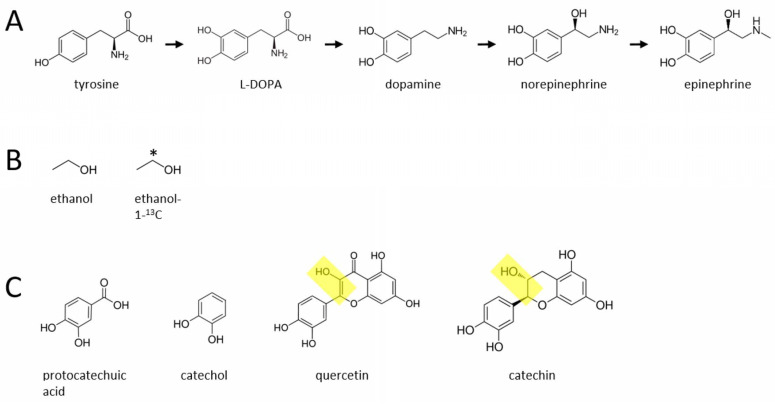
Canonical pathway for the endogenous synthesis of catecholamines and possible additional pathways involving ethanol. This pathway was described over 70 years ago, and posits that there is a single, principal biosynthetic pathway in mammals that converts the amino acid tyrosine to dopamine (via L-DOPA), and subsequently to norepinephrine and epinephrine (**A**). The middle portion of the figure (**B**) shows the structure of C12 unlabeled ethanol (left) and the C13 labeled ethanol (right) proposed to be used here. The * indicates where the C13 is located within the molecule. (**C**) Various plant-derived phenylic molecules that may combine with ethanol in vivo to produce catecholamines. Yellow boxes highlight an ethanol-like moiety already present within the flavonoids quercetin and catechin, that could lead to biotransformation to catecholamines even in the absence of ethanol.

## Data Availability

Not applicable.

## Sample Availability

Samples of the compounds are not available from the authors.
